# Applying the behavior change wheel to ulcerative colitis self-management: a conceptual closed-loop behavioral support framework

**DOI:** 10.3389/fmed.2026.1857587

**Published:** 2026-08-14

**Authors:** Zheng-ri Zhu, Xue Zhao, Chen-chen Wang, Jia-yuan Ding, Ying He

**Affiliations:** 1 Department of Gastroenterology, Hongqi Hospital Affiliated to Mudanjiang Medical University, Mudanjiang, China; 2 Second Ward of General Surgery Department, Hongqi Hospital Affiliated to Mudanjiang Medical University, Mudanjiang, China

**Keywords:** behavior change wheel, chronic illness self-management, COM-B model, digital health, health psychology, medication adherence, self-efficacy, ulcerative colitis

## Abstract

**Introduction:**

Ulcerative colitis (UC) is a chronic inflammatory bowel disease characterized by recurrent relapse and remission, requiring effective patient-led self-management. However, current self-management interventions often lack a systematic behavioral framework to support long-term behavior change.

**Methods:**

This perspective article proposes a behaviorally informed self-management framework for UC based on the Behavior Change Wheel (BCW). The framework was developed by integrating clinical guidelines, published literature, behavioral theory, and clinical observations. The Capability, Opportunity, Motivation-Behavior (COM-B) model was applied to identify behavioral barriers and guide intervention design.

**Results:**

The proposed framework integrates five key self-management behaviors, including medication adherence, dietary management, symptom monitoring, stress management, and regular follow-up. These behaviors are linked to COM-B-based barrier assessment, BCW intervention functions, behavior change techniques, blended delivery pathways, and an iterative closed-loop feedback system involving behavioral, clinical, psychological, and process monitoring.

**Discussion:**

The BCW-guided framework provides a theoretically grounded approach for developing individualized and adaptive self-management support in UC. Although the model remains conceptual and requires empirical validation, it may offer a structured pathway for future behavioral interventions and multidisciplinary chronic disease management.

## 1 Introduction

Ulcerative colitis (UC) is a chronic inflammatory bowel disease characterized by an unpredictable pattern of relapse and remission (1, 2). This fluctuating disease course imposes a persistent physical, psychological, and economic burden on patients (3). Although pharmacotherapy remains essential for inducing and maintaining remission, its limitations—such as variable treatment response, potential side effects, and high costs—underscore the importance of patient-led self-management (4, 5). Effective self-management enables patients to monitor symptoms, adhere to medications, regulate diet, manage stress, and recognize early signs of disease flare (6, 7). However, existing self-management interventions for UC are often not grounded in robust behavioral theory and provide insufficient support for long-term behavior change, thereby limiting their clinical effectiveness and generalizability (8).

From a psychological perspective, effective UC self-management depends on the interaction of cognitive, affective, motivational, and environmental mechanisms rather than on knowledge acquisition alone (9). Psychological constructs such as illness perceptions, self-efficacy, anxiety, depression, coping strategies, behavioral habits, and intention–behavior gaps may substantially influence patients' ability to sustain long-term self-management routines (10). Moreover, because UC symptoms fluctuate over time, patients are often required to repeatedly adapt behavioral strategies in response to changing physical conditions, emotional stressors, and social circumstances (11). Consequently, self-management failure should not be interpreted merely as insufficient disease knowledge, but rather as a multidimensional difficulty in behavioral regulation, self-regulatory maintenance, and adaptive coping (9).

The Behavior Change Wheel (BCW) is a comprehensive, theory-driven framework designed to develop behavior change interventions (12). At its core lies the Capability, Opportunity, Motivation-Behavior (COM-B) model, which proposes that any behavior results from the dynamic interaction of three essential components: capability (the psychological and physical ability to perform the behavior), opportunity (external environmental factors that enable or prompt the behavior), and motivation (automatic and reflective processes that initiate and sustain behavior) (12, 13). Surrounding the COM-B model, the BCW identifies nine intervention functions (e.g., education, persuasion, incentivization, training, enablement) and seven policy categories (e.g., communication, service provision) that directly link to specific behavioral determinants (12).

Compared with behavior theories that primarily focus on individual cognition or intention formation—such as the Health Belief Model or the Theory of Planned Behavior—the COM-B framework provides a more operational and intervention-oriented structure by explicitly linking behavioral determinants to modifiable intervention strategies (12). This multidimensional perspective is particularly relevant to UC self-management, where sustained behavioral adherence depends not only on knowledge acquisition or behavioral intention, but also on emotional adaptation, healthcare accessibility, social support, environmental opportunity, and long-term motivational reinforcement (12, 14, 15). Moreover, UC self-management behaviors are inherently dynamic and interdependent, requiring patients to continuously adapt behavioral routines in response to fluctuating symptoms and psychosocial stressors (15). In this context, the BCW framework offers a more comprehensive and clinically actionable approach for integrating behavioral diagnosis with intervention planning within a unified systematic model.

Within this conceptual framework, the proposed “closed-loop feedback” mechanism represents a dynamic and adaptive behavioral support process rather than a one-time educational intervention. Specifically, patient-generated behavioral and clinical information—including symptom reports, medication adherence patterns, self-monitoring frequency, and inflammatory indicators—is continuously collected, reassessed, and translated into tailored feedback to guide subsequent behavioral adjustment and intervention refinement (16, 17). The feedback process may be delivered through automated digital prompts, clinician-mediated behavioral coaching, or hybrid feedback pathways that combine digital and professional support. Importantly, this iterative structure enables continuous behavioral reassessment and self-regulatory reinforcement, thereby supporting long-term behavioral maintenance and adaptive self-management in response to the fluctuating clinical course of UC (17, 18).

The BCW has been successfully applied to various chronic diseases, demonstrating its usefulness in developing systematic and replicable behavior change interventions (19). Nevertheless, its application to UC self-management remains notably limited (8). Previous self-management interventions for UC and inflammatory bowel disease (IBD) have largely focused on patient education, telemedicine, medication adherence, symptom monitoring, or digital follow-up (20). Although these approaches have demonstrated varying degrees of clinical benefit, they have generally been developed pragmatically, with limited use of an explicit behavioral design framework (20, 21). Accordingly, behavioral determinants are often assessed in isolation, intervention components are seldom systematically linked to underlying behavioral mechanisms, and adaptive behavioral support across the disease course remains insufficiently integrated (20–22). Despite the recognized need for structured self-management support in UC, no existing intervention has systematically applied the full BCW framework to address the unique behavioral challenges faced by this population (8, 23). Current interventions typically target isolated behaviors without adequately addressing the interdependent roles of capability, opportunity, and motivation as defined by the COM-B model (12). By contrast, the present framework adopts the complete BCW methodology, integrating COM-B-guided behavioral diagnosis, explicit mapping of intervention functions and behavior change techniques, and an iterative closed-loop feedback strategy within a unified conceptual framework (20, 21). This theoretically coherent approach is intended to improve intervention reproducibility, facilitate individualized behavioral support, and promote sustainable long-term self-management. Consequently, a clear theoretical rationale and practical implementation pathway for a BCW-based self-management intervention in UC are lacking.

**Figure 1:**
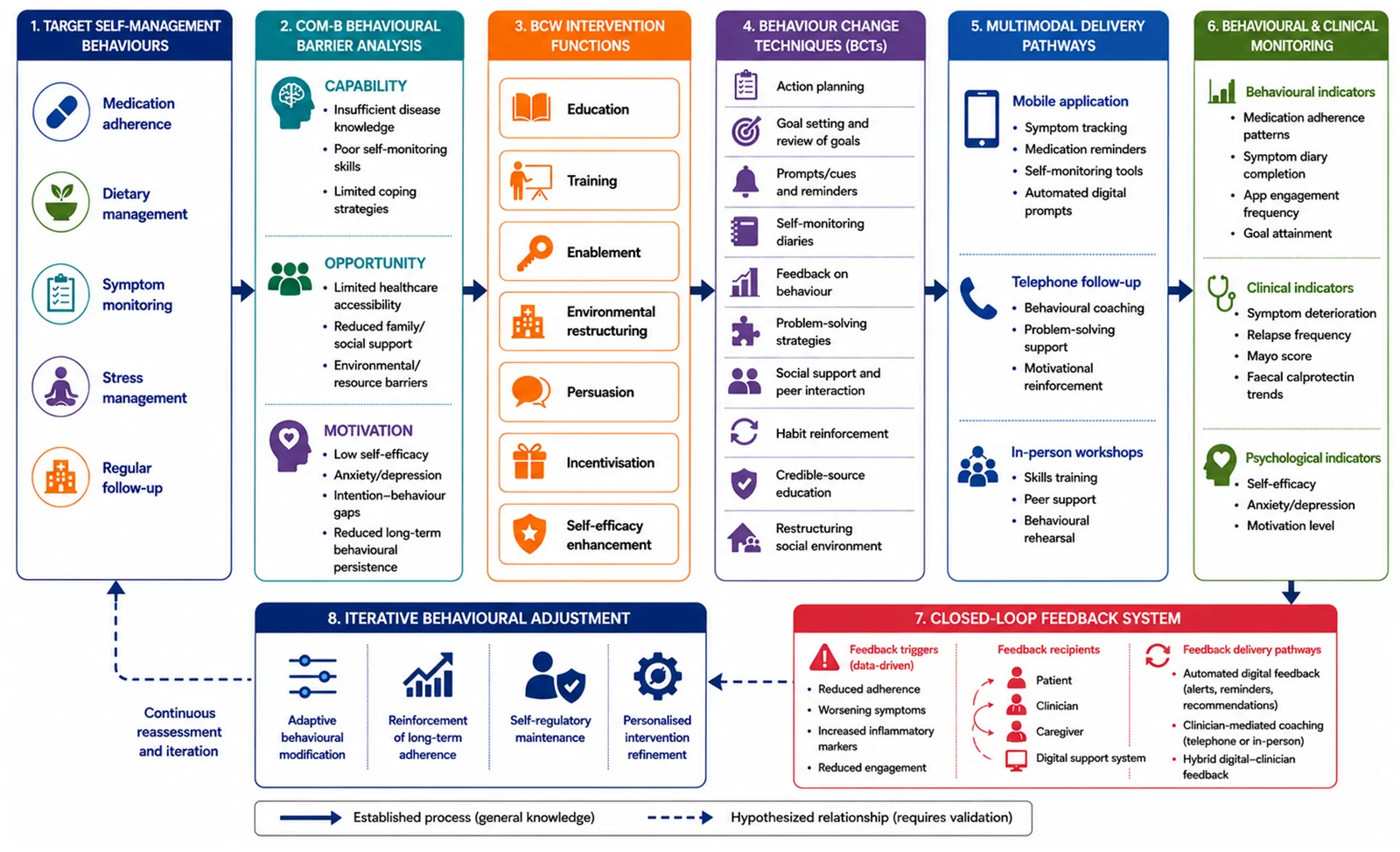
Conceptual closed-loop behavior support framework for ulcerative colitis self-management based on the bahavior change wheel.

The purpose of this perspective article is therefore to propose a logically coherent framework for constructing a BCW-guided self-management framework for UC (Figure 1). Specifically, this article outlines the theoretical logic underpinning the model, identifies its core content components, and describes practical implementation strategies derived directly from the BCW. By bridging the gap between behavioral science theory and clinical practice, this perspective article aims to inform the development of more behaviorally informed self-management programs tailored to individuals living with UC.

## 2 Theoretical framework construction: from COM-B to analysis of UC self-management behaviors

### 2.1 Defining target behaviors for UC self-management

UC self-management includes several related behaviors that patients need to perform regularly to control the disease and improve quality of life (24, 25). Based on clinical guidelines and existing research, five target behaviors are identified as core components: medication adherence (taking prescribed drugs at correct doses and times), dietary management (recognizing and avoiding foods that trigger symptoms), symptom monitoring (detecting early signs of disease relapse), stress management (using coping strategies to reduce psychological distress), and regular follow-up (attending scheduled clinical appointments) (24–26). Together, these behaviors influence disease outcomes and form the basis for further behavioral analysis.

### 2.2 Behavioral barrier analysis using the COM-B model

The COM-B model provides a structured approach to identifying barriers that prevent patients from performing target behaviors (12). Regarding capability, many patients lack sufficient disease-related knowledge (27). For example, they may misunderstand the chronic nature of UC or the need for maintenance therapy (28). They may also have poor self-monitoring skills, such as keeping symptom diaries or noticing small changes in stool frequency and consistency (29). In terms of opportunity, external environmental factors play a key role (30). Limited family support can reduce practical help with diet and medication routines (30, 31). Poor access to healthcare services—such as long travel distances or a shortage of specialists—hinders regular follow-up (30). In addition, limited access to reliable health information through digital or community channels restricts patients' ability to make informed decisions (27, 30). Regarding motivation, psychological and cognitive barriers are common. Disease-related cognitive biases, such as fatalistic beliefs or denial of illness severity, reduce engagement in self-management (32). Low self-efficacy, or lack of confidence in managing daily challenges, further undermines sustained behavior change (33, 34). Emotional disorders, particularly anxiety and depression, are frequent in UC and can directly weaken motivation to adhere to treatment or monitor symptoms (35).

### 2.3 Matching BCW intervention functions to identified barriers

Based on the COM-B analysis, specific intervention functions from the BCW can be matched to each type of barrier (Table 1). For capability deficits (knowledge and skills), education (providing information about UC and its management) and training (practicing self-monitoring and dietary decision-making) are suitable (12). For opportunity limitations (social and environmental factors), enablement (improving access to healthcare resources and social support) and environmental restructuring (changing physical or social contexts, such as using reminder systems or involving family members) are appropriate (12). For motivation deficits (cognitive and emotional factors), persuasion (using communication to address cognitive biases), incentivization (rewarding positive behaviors), and enablement (enhancing behavioral capability and self-efficacy through goal-setting, feedback, and supportive behavioral strategies) are relevant (12, 36). This systematic matching process provides the theoretical foundation for designing the subsequent framework.

**Table 1:** Mapping COM-B barriers to BCW functions, behavior change techniques, and delivery strategies in ulcerative colitis self-management.

Target behavior	COM-B domain	Barrier	BCW intervention Function	Behavior change techniques	Delivery mode	Evaluation indicators
Medication adherence	Capability	Insufficient disease knowledge	Education; Training	Action planning; credible-source education; medication reminders	Mobile app; telephone coaching	Adherence questionnaires; medication-taking consistency
Dietary management	Opportunity	Limited family/ social support	Enablement; Environmental restructuring	Family involvement; restructuring social environment; peer support	Workshops; caregiver education	Dietary adherence; symptom-trigger recognition
Symptom monitoring	Capability	Poor self-monitoring Skills	Training	Self-monitoring diary; prompts/cues; feedback on behavior	Mobile application	Symptom recording frequency; app engagement
Stress management	Motivation	Anxiety and emotional distress	Persuasion; Enablement	Motivational interviewing; problem solving; coping skills training	Telephone coaching; workshops	Anxiety/self-efficacy scales
Regular follow-up	Opportunity	Healthcare access barriers	Environmental restructuring	Telemedicine support; appointment reminders	Telemedicine platform	Follow-up attendance rate
behavioral maintenance	Motivation	Reduced long-term behavioral persistence	Incentivisation; Self-efficacy support	Goal setting; review of goals; habit reinforcement	Hybrid delivery model	Behavioral maintenance and retention

BCW, behavior change wheel; COM-B, capability, opportunity, motivation–behavior model.

To improve operational specificity, the present framework incorporates concrete behavior change techniques aligned with the intervention functions of the BCW (12). For example, action planning and goal-setting strategies can support medication adherence (37). Prompts, cues, and self-monitoring tools can facilitate symptom tracking and habit formation (37). Problem-solving and motivational feedback can strengthen behavioral persistence when disease conditions fluctuate, while social support and restructuring of the social environment can improve long-term behavioral maintenance (38, 39). Furthermore, education from credible sources and regular review of behavioral goals can reinforce self-efficacy and adaptive self-regulation (40). These techniques were included to translate abstract behavioral functions into more clinically actionable intervention components suitable for self-management in UC.

### 2.4 Framework development considerations

The proposed framework was conceptually developed by integrating three main sources: (1) international clinical guidelines for managing UC, (2) published literature on self-management in IBD and behavioral intervention development, and (3) the authors' clinical observations of common behavioral barriers encountered in routine practice (11, 41).

Because the present article is intended as a conceptual perspective rather than a systematic evidence synthesis, a formal systematic review was not undertaken. Instead, the evidence informing framework development was identified through narrative searches of PubMed, Web of Science, and international guideline repositories using combinations of keywords including “ulcerative colitis,” “inflammatory bowel disease,” “self-management,” “behavior change,” “COM-B,” and “Behavior Change Wheel.” Priority was given to international clinical guidelines, systematic reviews, behavioral intervention studies, and implementation science literature published in English to ensure that the proposed framework was grounded in contemporary evidence and established behavioral theory.

The five target behaviors were selected because they consistently appear across both guideline recommendations and self-management literature as core determinants of disease control and long-term management (37). Behavioral barriers associated with these target behaviors were subsequently synthesized through conceptual mapping using the COM-B model together with the principles of the Theoretical Domains Framework, thereby providing a structured theoretical basis for linking behavioral determinants to corresponding intervention functions (12, 13). Although formal patient interviews or Delphi consensus procedures were not conducted, the present model is intended as a hypothesis-generating conceptual framework to guide future empirical research on intervention development and validation.

## 3 Framework design: a BCW-based self-management program for UC

### 3.1 Intervention goals and principles

The main goal of this intervention is to improve patients‘ ability to change their behaviors when managing UC. To reach this goal, the intervention follows three core principles: stage-based progression (providing content that matches patients' readiness to change), individualization (adjusting strategies to each patient's specific barriers and preferences), and actionability (offering concrete and practical tasks that can be easily done in daily life) (42, 43). These principles aim to improve the feasibility and real-world applicability of the intervention.

### 3.2 Intervention content and delivery pathways (organized by BCW layers)

Based on the COM-B analysis and matched intervention functions, the content of the intervention is organized around the three BCW layers: capability, opportunity, and motivation (12), with each domain linked to corresponding BCW intervention functions and behavior change techniques.

For capability, the intervention strengthens disease knowledge and practical self-management skills through structured education, symptom self-monitoring training, personalized action planning, medication reminder systems, and digital prompts (12, 37, 44, 45). Together, these components enhance patients' capacity to perform essential self-management behaviors in routine daily life.

For opportunity, the framework incorporates peer support, family engagement, remote follow-up, and structured problem-solving strategies to strengthen the social and environmental conditions that facilitate sustained self-management (12, 31, 45–47). These components improve access to behavioral support while promoting continuity of care beyond routine clinical encounters.

For motivation, the framework integrates motivational interviewing, personalized goal-setting, behavioral feedback, self-reward mechanisms, and habit reinforcement strategies to strengthen behavioral engagement and adaptive self-regulation (48–50). These approaches are designed to sustain behavior change despite fluctuations in disease activity and everyday challenges.

Collectively, these intervention components translate the behavioral diagnosis into practical implementation strategies while maintaining explicit alignment between identified behavioral barriers and corresponding intervention functions.

### 3.3 Intervention format and duration

The proposed intervention adopts a blended delivery model that combines a mobile application (providing educational resources, reminders, and self-monitoring tools), telephone follow-up (offering personalized coaching and behavioral support), and in-person workshops (facilitating practical skills training and peer interaction) (45, 51, 52). The program is conceptually organized into an initial intensive phase followed by a maintenance phase, although the duration of each stage remains provisional and requires empirical validation.

This blended delivery strategy was selected to maximize accessibility, continuity of care, and sustained patient engagement by integrating complementary intervention modalities across different healthcare settings (53–55). Its flexible design also enables behavioral support to be maintained throughout the fluctuating course of UC while accommodating individual preferences and variations in healthcare resources.

## 4 Implementation and evaluation strategies

### 4.1 Considerations for implementation feasibility

Successful implementation of a BCW-based self-management intervention for UC depends on two main factors: integration into existing healthcare systems and strategies to reduce patient non-adherence and dropout.

For healthcare system integration, a multidisciplinary team approach is recommended (56). This team should include gastroenterology nurses (who provide patient education and follow-up), IBD specialists (who offer clinical oversight and treatment decisions), and behavioral health experts (who address psychological barriers such as anxiety or low motivation) (56, 57). A practical way to integrate the intervention is to embed it into routine outpatient services, with clearly defined roles and coordinated care pathways (58).

To manage the risks of poor adherence and patient dropout, several strategies can be used (59). These include setting clear expectations at enrolment, offering flexible communication channels (such as telephone, mobile application, or in-person visits), and giving regular positive feedback to keep patients engaged (59). In addition, early identification of patients at high risk of non-adherence—through baseline measures of motivation or past dropout history—allows for targeted retention efforts, such as extra coaching calls or simpler task requirements (60).

### 4.2 Framework adaptation across patient populations

Although the proposed framework offers a general behavioral structure, its implementation should be tailored to patient characteristics. In patients with active disease, greater emphasis should be placed on symptom monitoring, medication adherence, and timely clinical communication, whereas those in remission may derive more benefit from behavioral maintenance and relapse prevention (20, 61, 62). Newly diagnosed patients typically require intensive education and capability-building support, while individuals with long-standing disease may benefit more from motivational reinforcement and habit optimization (61). Older adults may need simplified digital platforms and increased caregiver involvement, whereas younger patients often engage more readily with mobile health technologies (63, 64). Furthermore, intervention intensity and communication strategies should be adapted to health literacy, socioeconomic resources, and healthcare access, so as to maximize feasibility, equity, and long-term behavioral engagement (20, 63, 64).

### 4.3 Recommended framework for evaluation indicators

A comprehensive evaluation of the intervention should cover four domains. At the behavioral level, validated tools for measuring self-management behaviors should be used, such as the IBD Self-Efficacy Scale or standard medication adherence questionnaires (34). At the clinical level, key outcome measures include disease activity measured by the Mayo score, fecal calprotectin levels as an objective marker of intestinal inflammation, and the frequency of relapse during follow-up (65). At the psychological and behavioral level, assessments should measure self-efficacy, illness perceptions, and motivational levels—the last of which can be assessed using a questionnaire based on the COM-B model (66, 67). For process evaluation, indicators should include participant engagement (for example, attendance at sessions or login frequency to the mobile application), satisfaction ratings, and intervention fidelity (the degree to which the intervention is delivered as planned) (68).

### 4.4 Recommendations for study design

Although the randomized controlled trial (RCT) remains the gold standard for establishing cause-and-effect relationships (69), a perspective article may suggest alternative designs that balance internal validity with practical applicability. A pragmatic cluster RCT, in which existing clinical units (such as outpatient clinics or hospital departments) are randomized instead of individual patients, can better reflect real-world conditions and reduce the risk of contamination between intervention and control groups (70). Alternatively, a mixed-methods design—combining quantitative outcome measures with qualitative interviews or focus groups—can provide deeper insight into how the intervention works and identify contextual factors that affect implementation success (71). It should be noted that the temporal structure of the proposed intervention is conceptual and intended to guide future study design, rather than to define a fixed protocol at this stage.

## 5 Discussion

### 5.1 Unique value of the BCW framework in UC self-management

Applying the BCW to UC self-management provides a systematic behavioral framework that extends beyond conventional education-based interventions (12, 72). Rather than targeting isolated behaviors, the proposed framework integrates behavioral assessment, intervention planning, and continuous behavioral support within a unified conceptual structure.

Compared with previously reported UC and IBD self-management interventions, which have primarily focused on patient education, symptom monitoring, telemedicine, or individual behavioral components, the proposed framework adopts a more comprehensive behaviorally informed design (12, 20, 21). Behavioral barriers are systematically identified using the COM-B model before intervention planning, enabling intervention functions and behavior change techniques to be explicitly matched to specific behavioral determinants. This transparent design process strengthens the theoretical coherence of the framework while facilitating reproducibility and future implementation. The principal distinctions between representative UC/IBD self-management interventions and the proposed BCW-guided framework are summarized in Table 2.

**Table 2:** Comparison of Representative UC/IBD Self-Management Interventions and the Proposed BCW Framework.

Comparison domain	Representative UC/IBD self-management interventions	Proposed BCW framework	Added value
Theoretical basis	Limited or inconsistent use of behavioral theory	Full integration of BCW and COM-B	Theory-driven intervention design
Behavioral assessment	Focus on isolated behavioral problems	Systematic COM-B behavioral diagnosis	Comprehensive barrier identification
Intervention design	Empirical or behavior-specific strategies	Explicit mapping from barriers to intervention functions and BCTs	Transparent and reproducible design
Behavior change techniques	Implicit or variably applied	Explicitly selected and operationalized	Improved implementation fidelity
Personalization	Standardized interventions	Barrier-based tailored support	Individualized behavioral management
Feedback strategy	Periodic education or follow-up	Continuous closed-loop feedback	Dynamic behavioral adaptation
Delivery approach	Single intervention modality	Blended digital and face-to-face delivery	Multi-component behavioral support
Clinical implementation	Stand-alone educational programmes	Integrated multidisciplinary framework	Greater scalability and clinical applicability
Long-term objective	Improve specific behaviors	Sustain adaptive self-management	Enhanced long-term behavioral maintenance

BCW, behavior change wheel; COM-B, capability, opportunity, motivation–behavior; BCTs, behavior change techniques; UC, ulcerative colitis; IBD, inflammatory bowel disease.

Another distinguishing feature is the incorporation of an iterative closed-loop feedback mechanism. Continuous behavioral reassessment enables intervention strategies to be dynamically refined according to changes in disease activity, behavioral performance, and individual patient needs, thereby extending beyond the predominantly one-directional educational or monitoring approaches reported in previous UC self-management programs (12, 20, 21).

Collectively, these features enhance the transparency, reproducibility, scalability, and potential clinical applicability of the proposed framework, providing a robust conceptual foundation for future behaviorally informed self-management interventions in UC.

### 5.2 Innovations and limitations of this framework

The principal innovation of the proposed framework lies in its systematic translation of behavioral theory into an integrated self-management combine COM-B-guided behavioral diagnosis, explicit mapping of BCW intervention functions and behavior change techniques, and an adaptive closed-loop feedback process within a single implementation framework. By explicitly linking behavioral determinants to intervention components, the framework provides a transparent, reproducible, and theoretically grounded foundation for future intervention development and empirical evaluation.

However, several limitations should be acknowledged. First, the framework remains conceptual and has not yet been empirically validated in large UC populations. Consequently, future studies are required to determine whether the proposed behavioral diagnosis and intervention strategies improve long-term behavioral and clinical outcomes (8). Moreover, poor self-management in UC may arise not only from behavioral capability deficits but also from medication-related adverse effects, limited health literacy, socioeconomic disadvantage, occupational constraints, cultural dietary practices, stigma, and reduced patient–clinician trust. These factors extend beyond the COM-B framework and may require complementary theoretical perspectives (27, 30, 61, 73). Second, implementation of this multi-component intervention—including development of digital platforms, multidisciplinary workforce support, and remote follow-up systems—may require substantial organizational and financial resources (20, 21). Future implementation research should therefore evaluate its cost-effectiveness and scalability in routine clinical practice (74). Finally, integrating digital health technologies introduces additional challenges, including digital exclusion among older adults and individuals with limited technological literacy, alert fatigue, privacy and data security concerns, self-monitoring burden, and overreliance on patient-generated data. These issues may influence intervention acceptability, feasibility, and long-term adherence across different healthcare settings (75–77).

### 5.3 Implications for future research and clinical practice

This perspective suggests several directions for future research and clinical practice. First, multicentre validation studies are recommended to test the effectiveness and generalizability of the proposed intervention across different UC populations and healthcare systems (78). Second, digital health technologies, especially mobile health (mHealth) tools, can be added to strengthen behavioral support (79). Features such as real-time symptom tracking, automated medication reminders, and personalized feedback can help patients manage their condition between clinic visits (80). Third, we recommend incorporating the BCW framework into the standardization of IBD nursing care pathways. By embedding behavioral science into routine clinical protocols, healthcare systems can more consistently meet patients' self-management needs and improve long-term disease outcomes (12).

## 6 Summary

The self-management framework based on the BCW theory provides a structured framework for supporting self-management behaviors in patients with UC. Using the COM-B framework—capability, opportunity, and motivation—this model goes beyond traditional education-based methods by addressing the multiple barriers that limit effective self-management. Key target behaviors include medication adherence, dietary management, symptom monitoring, stress management, and regular follow-up. These are linked to matched intervention functions such as education, training, enablement, environmental restructuring, persuasion, and incentivization. The proposed intervention uses a blended delivery format that combines a mobile application, telephone follow-up, and in-person workshops. Its conceptual framework consists of an initial intensive phase followed by a maintenance phase. The optimal duration of each phase requires validation through future studies. Evaluation strategies cover behavioral, clinical, psychological, and process indicators.

Although the proposed framework is theoretically informed and conceptually structured, it remains a hypothesis-generating model that has not yet been empirically validated in clinical populations. Moreover, the acceptability, feasibility, and practical implementation of its different components may vary across healthcare systems, patient populations, and resource settings. Therefore, theoretical coherence alone should not be interpreted as evidence of behavioral or clinical effectiveness. Further multicentre studies, pragmatic implementation research, and cost-effectiveness analyses are needed to evaluate the real-world applicability and clinical impact of the proposed framework. Accordingly, the present model should be viewed primarily as a conceptual foundation—intended to inform future behavioral intervention development and empirical investigation in UC self-management—rather than as a validated clinical program.

## Data Availability

The original contributions presented in the study are included in the article/supplementary material, further inquiries can be directed to the corresponding author.
